# Capacitance Measurements for Evaluating Electrochemical Double‐Layer Models and Potentials of Zero Charge: A Reassessment

**DOI:** 10.1002/cphc.202401088

**Published:** 2025-05-25

**Authors:** Maximilian Schalenbach, Hermann Tempel, Rüdiger‐A. Eichel

**Affiliations:** ^1^ Institute of Energy Technologies (IET‐1): Fundamental Electrochemistry Forschungszentrum Jülich 52425 Jülich Germany; ^2^ Institute of Physical Chemistry RWTH Aachen University 52062 Aachen Germany

**Keywords:** cyclic voltammetries, differential capacitances, double‐layer capacitances, impedance spectroscopies, potentials of zero charges

## Abstract

Differential capacitances (DCAPs) derived from electrostatic Gouy–Chapman‐type models for electrochemical double layers (DLs) typically show valley‐, bell‐, or camel‐type profiles as a function of the potential, centered around the potential of zero charge. These DCAP profiles are routinely evaluated with measured potential dependencies of capacitances. Here, the influences of hydrogen evolution, oxygen reduction, and oxide formation on the potential dependence of the capacitance of a polished gold electrode are experimentally examined. These parasitic reactions are found to cause most of the potential‐dependent capacitance features that are typically attributed to intrinsic DL properties. With these insights, the historical development of the literature regarding the development of the theoretical framework in relation to capacitance measurements is critically reevaluated. As a result, drawbacks of the 100‐year‐old Gouy–Chapman theory for the DL are identified. Moreover, DCAPs as differences of electrostatic states are discussed as unable to portray measured capacitances that result from capacitive–resistive and dynamic charge displacements in the DL. Hence, the links between theories and experiments are critically assessed, motivating the need for more advanced atomistic models to adequately portray the DL.

## Graphical abstract

The electrochemical currents of oxide formation, hydrogen evolution reaction, and oxygen reduction reaction are shown to affect the potential dependencies of measured capacitances. Based on these findings, the routine approach of evaluating models for the electrochemical double layer and determining potentials of zero charge by capacitance measurements is critically assessed.

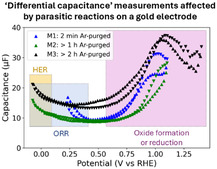

## 1 Introduction

The electrochemical double layer (DL) describes the charge arrangement at the electrochemical interface, in which electrode potentials locally disturb the even ion distributions of bulk electrolytes as determined by the electroneutrality condition.^[^ ^1^ ^]^ In this introduction, the historical development of DL models and measurement techniques is reviewed, aiming to discuss how the links between models and measurements were established. Based on this brief review of the DL fundamentals, models and experiments will be critically analyzed and reevaluated.

In 1879, Helmholtz first examined the electric potential transition between a metal and an electrolyte.^[^ ^2^ ^]^ His model for the electrochemical interphase as opposing and infinitely thin surface charge layers at both media led to the denotation of a DL of charges that face one another at the interface. This interpretation of the interface reflects Gauss's law of electrostatics, from which displaced mobile charge carriers at the surfaces can be derived to shield conductive bulks. Thus, in this model, the bulks of both touching conductive media, electrode and electrolyte, are free of macroscopic electric fields, respectively. Electrostatics describes stationary electric charges, which implies no charge exchange or currents. Hence, the electric field‐free bulks of the conductors can be alternatively reasoned by Ohm's law.

In the beginning of the 20th century, Gouy^[^ ^3^ ^]^ and Chapman^[^ ^4^ ^]^ independently developed an electrostatic DL model in which the basic physical principles of thermomechanical statistics (Boltzmann equation) and electrostatics (Poisson equation) to the electrolytic part of the DL^[^ ^5^ ^]^ have been combined. As a result, Helmholtz interpretation of infinitely thin surface charge layers has been softened by spatially spread distributions of potential and ion concentrations in the electrolyte.

**Figure 1:**
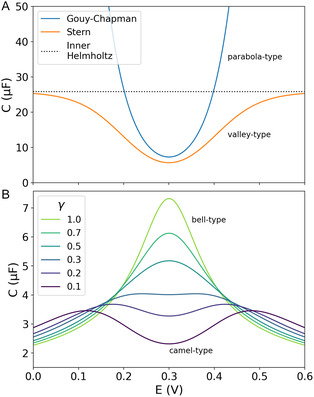
Capacitances of common double‐layer models as a function of the electrode potential, assuming a potential of zero charge of 0.3 V. A) Classical Gouy–Chapman and Stern theory, modeled based on another study.^[^ ^10^ ^]^ B) Lattice gas distribution models based on^[^ ^37^ ^]^ with a variation of compacity *γ* [8] as the degree of compressibility. This model can mimic the often‐reported camel‐to‐bell transition of measured capacitance profiles as a function of the electrolyte concentration.

Deviating the modeled charge density of the DL in the electrode potential leads to a capacitance, which is commonly referred to as differential capacitance (DCAP).^[^ ^6^, ^7^, ^8^, ^9^ ^]^ **Figure** **1**A shows the parabola‐like shape of the DCAP from the Gouy–Chapman model as a function of the electrode potential. The minimum capacitance of this relation represents the uncharged surface. The potential at this minimum is commonly referred to as the potential of zero charge (PZC).^[^ ^10^, ^11^, ^12^ ^]^ In 1924, Stern^[^ ^13^ ^]^ described the DL capacitance as a serial combination of the inner Helmholtz's capacitance $C_{\text{IH}}$ and the DCAP $C_{\text{diff}}$.

(1) $$C_{\text{Stern}}={\left(\frac{1}{C_{\text{diff}}}+\frac{1}{C_{\text{IH}}}\right)}^{-1}$$

Hence, Stern combined Helmholtz's model of a rigid “inner” DL with a diffusive “outer” layer.^[^ ^5^, ^9^, ^14^ ^]^ Figure 1A shows that Stern's extension limits the unphysical diverging capacitance of the parabola‐type DCAP of the Gouy–Chapman model, leading to a valley‐type capacitance as a function of the potential.

In 1929, the valley‐type DCAPs predicted by the classical models were compared to electrocapillary measurements with mercury,^[^ ^15^ ^]^ as direct capacitance measurements were not feasible at that time. Hereto, the Lippman Equation^[^ ^16^ ^]^ was used to estimate the surface charge density based on measuring the capillary of the mercury–electrolyte interface under a potential variation. By deviating the thus estimated charge densities after the measured electrode potential, the capacitance of the mercury electrode was determined. With the advances of electronic measurement devices, electric measurements of the capacitance as a function of the electrode potential were introduced in 1935.^[^ ^17^ ^]^ Sinusoidal potential variations were added to the applied electrode potential from which capacitances were extracted,^[^ ^18^ ^]^ showing good agreement with the capacitances derived from the electrocapillary measurements.^[^ ^17^, ^19^, ^20^ ^]^ The measured capacitance profiles (MCPs) agreed reasonably with calculated DCAPs. Based on these promising results, MCPs were commonly accepted to directly portray the DCAP from the electrostatic DL models.^[^ ^7^, ^18^, ^21^, ^22^ ^]^ Decades later, MCPs were routinely measured during cyclic voltammetry (CV) with a simultaneous sinusoidal perturbation,^[^ ^23^, ^24^ ^]^ nowadays typically denoted as alternating cyclic voltammetry (ACV).

Further extensions on the GCMs included different ion sizes or interactions between the metals and the electrolyte,^[^ ^25^, ^26^, ^27^ ^]^ showing asymmetry in the capacitance as a function of the potential. By introducing the lattice gas theory into the GCMs,^[^ ^28^, ^29^, ^30^, ^31^, ^32^ ^]^ camel‐ and bell‐type shapes of capacitance profiles could be simulated as shown in Figure 1B. Hence, a more detailed match of the DL theories to MCPs was achieved,^[^ ^33^, ^34^, ^35^, ^36^ ^]^ including the camel‐to‐bell‐type transition as a function of the electrolyte concentration that was already observed in the early works with the mercury electrode in NaF electrolytes.^[^ ^20^ ^]^ In these models, the packing density *γ* of the electrolyte represents a parameter to account for the electrolyte concentration.^[^ ^22^, ^37^ ^]^ Moreover, the effects of the solvent^[^ ^38^, ^39^, ^40^ ^]^ and the electronic structure of the electrode^[^ ^41^, ^42^ ^]^ were added to the classical models.

Critics of the GCMs are a too narrow estimation of the DL, the issues with strong electric fields that lead to dielectric breakdown, to name just a few.^[^ ^43^ ^]^ Modern atomistic models for the DL display a great chance to overcome the drawbacks of the classical models.^[^ ^44^, ^45^, ^46^, ^47^, ^48^, ^49^ ^]^ For instance, molecular dynamics simulations can show that the assumption of a dielectric continuum is not justified due to anisotropic arrangements of water molecules.^[^ ^50^ ^]^ Like the classical models, these modern models are also often evaluated with the measured camel‐ and bell‐type capacitances as a function of the potential.^[^ ^51^, ^52^, ^53^, ^54^, ^55^, ^56^ ^]^ The often used 10 mM aqueous electrolyte for MCPs means that an ion pair is dissolved in more than 5000 water molecules. As the DL charging is an electrodynamic process that results from the interaction of millions of moving ions. Including also the solvent molecules means that billions of constituents must be considered to precisely model the DL. This n‐body problem with such large numbers of atoms, spatial scales, and time scales for the ion rearrangement is challenging to model with modern atomistic models such as density functional theory or molecular dynamics techniques. Hence, the classical Gouy–Chapman‐type models still represent today's textbook knowledge of the electrochemical interface, which is justified by a very good agreement in comparison to experimental data.

In 1984, Brug reported that the response of the DL in impedance spectroscopy shows a constant phase with higher values than the −90° of a capacitance.^[^ ^57^ ^]^ He parameterized the DL by a constant phase element (CPE). In 1987, Wang reported that a CPE can be represented by a transmission line model of a semi‐infinite resistive–capacitive ladder network.^[^ ^58^ ^]^ This CPE‐type response of the DL was explained by the transmission line character that results from the electric penetration into the electrolyte and the associated ion displacement with its resistive damping.^[^ ^59^, ^60^ ^]^ In contrast, DCAPs of the electrostatic models describe transition between two static states by ignoring the dynamic process of the charge displacement in the DL. This obvious inconsistency between the observed CPE‐type character of the DL and the solely capacitive description of the electrostatic theories is unfortunately widely ignored in the community.

The aim of this study is to critically analyze MCPs regarding the impact of parasitic reactions like oxygen reduction, hydrogen evolution, and electrode oxidation. Gold, as the noblest of all metals,^[^ ^61^, ^62^ ^]^ is used as a model electrode for these experiments, allowing oxide free surface preparation by polishing. An aqueous sodium fluoride electrolyte with a concentration of 5 mM is used as the electrolyte, with its symmetric ions representing the literature standard for DCAP measurements.^[^ ^18^, ^20^, ^23^ ^]^ Features of MCPs that were previously associated with the intrinsic properties of the DL are here attributed to parasitic reactions, also showing that the surface preparation is an important factor that led to previous misinterpretations of MCPs on gold. The commonly accepted link between MCPs and DCAPs is reevaluated regarding the dynamics of the charge delocalization measured in the experiments but ignored in electrostatic DL theories. Published data on MCPs and PZCs are critically discussed and analyzed regarding parasitic reactions and the role of oxide layers. Hence, this study provides a critical view of electrostatic DL models that shall motivate the community to develop advanced atomistic models for the electrochemical interface.

## 2 Experimental Section

### 2.1 Measurement Protocol

**Figure 2:**
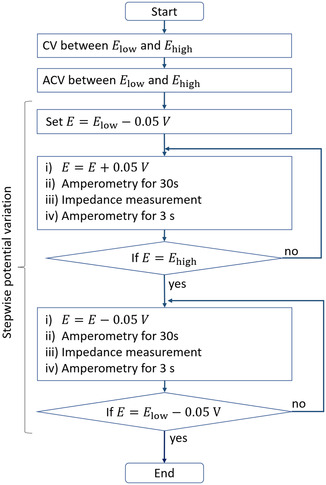
Flowchart of the measurement procedure used in this study.

**Figure** **2** shows the measurement protocol used in this study, in which initially, CV with three cycles and a scan rate of 10 mV s^−1^ is applied. The lower and upper margins of this measurement are represented by the low vertex potential $E_{\text{low}}$ and the high vertex potential $E_{\text{high}}$, respectively. Subsequently, ACV is conducted, using again a scan rate of 10 mV s^−1^ and a perturbation frequency of 1 Hz with an amplitude of 10 mV. The ACV measurement starts at $E_{\text{high}}$. After the CV and ACV measurement, a stepwise potential variation is employed that starts at $E_{\text{low}}$. In this procedure, amperometry is used to measure the current after a potential step. Afterward, an impedance measurement between 200 mHz and 200 kHz is conducted with an amplitude of 10 mV. Amperometry is applied again, before the next step starts with a potential variation. Starting from $E_{\text{low}}$, the potential is incrementally increased by 50 mV until $E_{\text{high}}$ is reached. Afterward, the direction of the potential step is reversed until $E_{\text{low}}$ is reached again.

### 2.2 Experimental Methods and Materials

The electrochemical three‐electrode cell used for the experiments in this study was reported in the literature.^[^ ^63^ ^]^ A cold‐rolled polycrystalline gold electrode (Junker Edelmetalle GmbH) was used as the working electrode, prepared by grinding with 4000 mesh SiC sandpaper (Struers) and then polished with 1 μm diamond particle solution (Struers) for 20 min on an automized polishing machine (Struers, Tegramin). A stamped fluoropolymer flat sealing of 0.5 mm thickness was used to confine the exposed geometric area of the gold plate exposed to the electrolyte to 0.78 cm^2^ (diameter of 1 cm). An Ag/AgCl reference electrode (Methohm) was used in a separated electrolyte compartment, while a platinum mesh counter electrode was employed, which was also placed in a separated electrolyte compartment. An electrolyte of NaF (99.99%, Thermo Fisher) dissolved in ultrapure water with a concentration of 5 mM was used for all presented measurements. The measured potential of the Ag/AgCl reference electrode was converted to the reversible hydrogen electrode (RHE) by adding 0.197 V for the offset of the reference electrode, while the pH was included by adding its value times 59 mV. The pH of the electrolyte was measured with a pH meter (Mettler Toledo, SevenCompact) to 7±0.5. Hence, the RHE values stated in this study were affected by an absolute error of ≈±0.03 V.

A biologic VMP‐3 potentiostat was used for all measurements. To reduce the amount of dissolved oxygen in the electrolyte, it was purged with argon using an immersed porous glass frit. The fine pores of the frit resulted in small bubbles that effectively removed oxygen. The entire cell was placed in a plastic bottle so that argon coming from the cell accumulated in the bottle, reducing the oxygen contamination from the environment. This plastic bottle was placed in a grounded metal container, which acted as a Faraday cage to eliminate electric stray fields.

### 2.3 Data Evaluation

The impedance evaluation of the data was discussed in a previous study.^[^ ^63^ ^]^ The Biologic potentiostat recorded the ACV measurement in the time domain with voltage and current in the resolution of 1 ms. From these time‐domain resolved data, the impedance amplitude and phase angle were calculated with the Python code supplied in the electronic supplementary information (ESI). In brief, the slope of the potential and current signal related to the scan rate of 10 mV s^−1^ was subtracted. The signal was smoothed, and the impedance was calculated based on the amplitude of the alternating current and the phase between the potential and current. The procedure to extract the capacitance from the stepwise potential variation is described in the ESI.

## 3 Results and Discussion

### 3.1 Measurements on the Polished Gold Electrode

The measurement protocol graphed in Figure 2 was applied three times in series to the polished polycrystalline gold electrode. First, the data were collected between 0.2 and 1.1 V versus RHE with the 5 mM NaF electrolyte that was previously purged for 2 min with argon, denoted as M1. Second, the electrolyte was purged for 1 h with argon and the electrochemical data were collected in between −0.1 and 1.1 V versus RHE, denoted as M2. Third, the protocol with the margins of −0.1 and 1.4 V was applied to the argon‐purged electrolyte, denoted as M3. All measurements were conducted without intermediate surface treatment or electrolyte exchange.

**Figure 3:**
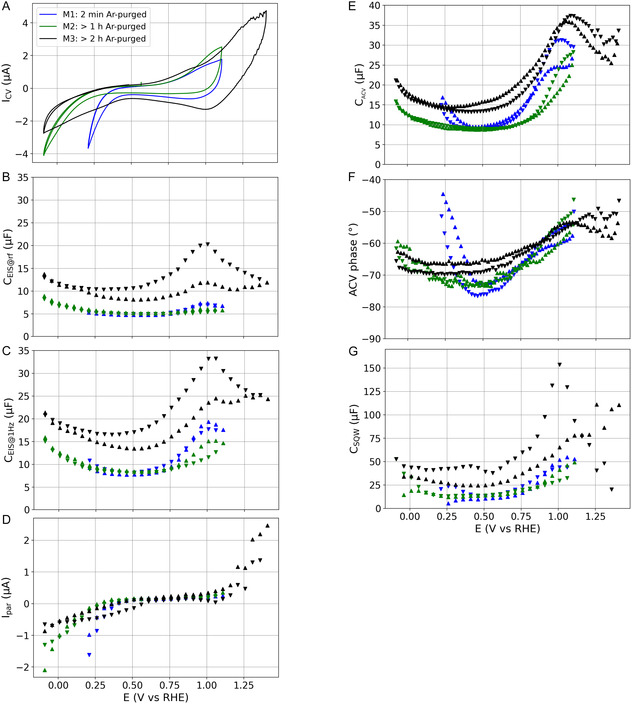
Data of the gold electrode in 5 mM NaF electrolyte obtained with three consecutive iterations of the measurement protocol from Figure 2. Blue: Measurement M1 (previous 2 min of argon electrolyte purging). Green: Measurement M2 with at least one hour of argon purging. Black: Measurement M3 with argon‐purged electrolyte. Triangles upwards: Data related to an increasing potential or ramp. Triangles downward: Down‐scan. A) CV measurements. B) Capacitances of impedance measurements at the relaxation frequency. C) Capacitances of impedance measurements at 1. D) Parasitic current at the end of each potential step measured by amperometry. E) The capacitance extracted from ACV measurements with a perturbation frequency of 1 Hz. F) The phase angle extracted from the ACV measurements. G) Capacitance from the stepwise potential variation of a SQW type.

**Figure** **3** shows the data recorded, comprising 1) CV measurements with the current $I_{\text{CV}}$ as a function of the electrode potential *E*. 2) The capacitance $C_{\text{EIS}@\text{RF}}$ is obtained via electrochemical impedance spectroscopy (EIS) at the relaxation frequency (RF). The capacitance at the RF was introduced in previous works as a reliable metric for surface area estimations.^[^ ^63^, ^64^ ^]^ The impedance spectra of the measurements are graphed in the electronic supporting information (ESI), showing that the RFs of the polished gold specimen in the used 5 mM NaF electrolyte ranged between 100 and 300 Hz. 3) The capacitance $C_{\text{EIS}@1\text{Hz}}$ equals the capacitive contributions to the EIS data at 1 Hz. 4) The parasitic electrochemical current $I_{\text{par}}$ is measured by amperometry after the impedance measurements (see Figure 2). 5) The capacitance $C_{\text{ACV}}$ is obtained from alternating cyclic voltammetry (ACV) measurements. 6) The phase angle of the ACV measurements, which indicates the balance of resistive and capacitive contributions to the sinusoidal perturbation. 7) Square wave (SQW) capacitances, which derived from the amperometry after the potential step of 0.05 V of the measurement protocol. Charge transfer reactions are known to increase the electrode capacitance,^[^ ^63^, ^65^ ^]^ which is discussed for the MCPs in the following.

### 3.2 Surface Oxidation

In Figure 3A, the CV data of all measurements show above 0.8 V drastically increasing currents, which indicate an oxidative charge transfer reaction. The standard potential of gold is reported as 1.52 V,^[^ ^66^ ^]^ which is calculated based on the thermodynamics of bulk properties. However, the potential at which its oxidation starts depends on the oxide species formed, while gold's hydroxide surface species^[^ ^67^ ^]^ are typically not considered for the thermodynamic calculation of the standard potential. The surface oxidation can also lead to dissolution,^[^ ^68^, ^69^ ^]^ which depends on the amount of dissolved species (as graphed in Pourbaix diagrams^[^ ^70^ ^]^). Moreover, different facets show different stabilities,^[^ ^71^ ^]^ while the surface thermodynamics are different from that of the bulk.^[^ ^72^ ^]^ Hence, classical thermodynamic data for the nobleness of gold can be understood as an estimation rather than a precise value. In an alkaline regime, the dissolution of polycrystalline gold is reported to start at 1.12 V versus RHE^[^ ^69^ ^]^ (with respect to the logarithmic data of the dissolution rate as a function of the electrode potential), while the detection limit of this measurement technique overestimates the potential at which the dissolution starts. In addition, the surface can oxidize prior to the dissolution process, so that the oxidative current above 0.8 V is most likely resulting from the oxidation of some parts of the polycristalline surface.

Measurement M3 shows a reduction reaction (valley at 1 V) during the descending branch, which originates from the electrochemical reduction of at least one of the different oxide species formed.^[^ ^73^ ^]^ The dissolution and recrystallization during the oxidation or reduction also tend to increase the electrochemically active surface area (ECSA) of polished electrodes.^[^ ^63^, ^65^ ^]^ However, non or weak conducting oxide layers tend to decrease the electrode capacitance.^[^ ^63^, ^65^, ^74^ ^]^ In case of conductive oxide layers, the capacitance increases due to the higher ECSA.^[^ ^63^, ^65^ ^]^ When reduced after oxidation, the surface changes are irreversible due to the preferential dissolution of different crystal sites^[^ ^75^ ^]^ and kinetic barriers to reduce the oxides. As a result, all capacitances (Figure 3B,C,E, and G) of M3 are larger than those of M1 or M2.

In Figure 3B, $C_{\text{EIS}@\text{RF}}$ (measured above 100 Hz) of M1 and M2 only slightly depend on the potential and the oxidation process with rather flat MCPs. In contrast, $C_{\text{EIS}@1\text{Hz}}$ in Figure 3C shows a significant increase above 0.8 V by the oxidation process, as charge transfer reactions increase the impedance toward low frequencies.^[^ ^63^, ^65^ ^]^ In the case of M3, $C_{\text{EIS}@\text{RF}}$ and $C_{\text{EIS}@1\text{Hz}}$ show significant enlargements, attributed to the ECSA increase by the surface oxidation and reduction. The distinct differences of the capacitances between the ascending and descending branch for measurement M3 show that the oxidation and reduction lead to an irreversible change of the surface.

The $C_{\text{ACV}}$ profile in Figure 3D shows more pronounced hysteresis of the M1 and M2 data than that of the $C_{\text{EIS}@\text{RF}}$ and $C_{\text{EIS}@1\text{Hz}}$ data. Moreover, the enlargements of the capacitances from 0.8 to 1.1 V are steeper for $C_{\text{ACV}}$ than those for $C_{\text{EIS}@\text{RF}}$ and $C_{\text{EIS}@1\text{Hz}}$. These differences are ascribed to the more pronounced dynamics of the oxidation process due to the faster potential variation of the AVC technique in contrast to the steady state‐like perturbations during the impedance measurements. These dynamics of the phase transition also cause a more pronounced hysteresis of $C_{\text{ACV}}$ between the ascending and descending branches than those of $C_{\text{EIS}@1\text{Hz}}$. The phase angle of the ACV data in Figure 3F shows a similar profile as that of the $C_{\text{ACV}}$, indicating that partly Ohmic processes are responsible of the MCPs.

### 3.3 ORR and HER

The ORR is characterized by a reversible potential of 1.23 V versus RHE. In near‐neutral electrolytes, the ORR on gold shows sluggish kinetics, so that large overpotentials are required to achieve significant currents.^[^ ^76^ ^]^ In Figure 3A, the CV data of measurement M1 shows below 0.4 V negative currents, which are drastically reduced with the argon‐purged electrolyte of measurement M2. Hence, the reduction current of M1 is attributed mainly to the reduction of dissolved oxygen, while sluggish kinetics suppress the reduction currents at higher potentials. For M2, a significant reduction process starts below 0.2 V, which can also be influenced by the reduction of the surface oxides that were formed above 0.8 V. This process is even more pronounced for M3, where higher apex potentials led to an increased surface oxidation. The HER is expected to start already slightly above 0 V, as the reversible potential of the HER increases toward lower amounts of dissolved hydrogen (Nernst equation).^[^ ^77^ ^]^ Toward lower potentials, the contributions of the HER to the measured currents increase.

In Figure 3B, the capacitance $C_{\text{EIS}@\text{RF}}$ (probed at frequencies between 100 and 300 Hz) of measurement M1 is not significantly influenced by the ORR or other potential dependent features. In contrast, the capacitance $C_{\text{EIS}@1\text{Hz}}$ (Figure 3C) is significantly affected by the ORR as the contributions of charge transfer reactions to the impedance increase toward low frequencies.^[^ ^63^, ^65^ ^]^ In the case of M2 and M3, significant increases of $C_{\text{EIS}@\text{RF}}$ and $C_{\text{EIS}@1\text{Hz}}$ below 0.1 V are observed due to the HER. For the case of the platinum electrode, the oxygen adsorption was reported to affect the pseudocapacitance less than hydrogen adsorption,^[^ ^60^ ^]^ which was attributed to the slower kinetics of the oxygen adsorption. These findings are transferable to the pseudocapacitances associated with the hydrogen‐ and oxygen‐related charge transfer reactions on the gold electrode. Hence, the different effect of the HER and OER on the frequency‐dependencies of the capacitive contributions on the impedance can be explained.

Figure 3D shows the parasitic currents (measured at the end of each potential step by amperometry), which feature in the case of M1 a significant increase below 0.4 V that can be attributed to the ORR. Figure 3E shows the capacitances $C_{\text{ACV}}$ that were extracted from the ACV measurements at a perturbation frequency of 1 Hz. Below 0.5 V, $C_{\text{ACV}}$ shows a similar profile as $C_{\text{EIS}@1\text{Hz}}$. Both capacitances are probed at 1 Hz, yet the capacitance $C_{\text{ACV}}$ is obtained during a perturbated linear potential variation, while $C_{\text{EIS}@1\text{Hz}}$ is obtained at a perturbated constant potential. These different measurement conditions for the capacitance do not affect the trends for the ORR or HER contributions to the measured capacitances.

The phase angle of the ACV signal in Figure 3F increases by the contributions of the ORR in measurement M1. The resistive contributions of the ORR shift the phase angle toward higher values. The HER shows a less pronounced effect on the phase angle as it affects the potential perturbation more pseudocapacitive as Ohmic.^[^ ^60^ ^]^ For measurement M1, $C_{\text{SQW}}$ in Figure 3G becomes negative below 0.5 V due to the parasitic contribution of the ORR (like CV data from Figure 3A). The data of M1 and M2 show below 0.1 V differences between the ascending and descending branch of the potential variation, which is ascribed to a hysteresis of the hydrogen or bubble coverage. Concluding, the increase of the phase angle toward the lowest potentials indicate parasitic currents that eventually increase the capacitances.

To summarize, the parasitic currents related to the ORR, HER, oxide layer formation, and oxide layer reduction increase the capacitances. Measurement M2 represented the case of a mostly pristine gold surface in argon‐purged electrolyte. The capacitance data of measurement M2 shows between 0.1 and 0.8 V a flat MCP without any clear hint for valley‐, camel‐, or bell‐type features. At lower or higher potentials, the impact of the charge transfer reactions and pseudocapacitances leads to deviations from this flat MCP.

### 3.4 The Role of the Electrode Pretreatment

In the following, the above‐presented measurements are used to critically evaluate the literature data on MCPs and PZCs. The ORR and HER can increase capacitances toward low potentials, from which the left branch of a valley‐type MCP can originate. Moreover, the above‐presented measurements showed that dissolved oxygen shifts the left branch rightward. Experimental works presented in the literature often do not comment on how or if dissolved oxygen in the electrode was treated,^[^ ^24^, ^78^ ^]^ which may mean that a lot of these data is obtained with oxygen‐containing electrolytes. Above, a porous glass frit was used for argon purging, which is more effective in removing oxygen than purging the standard approach with a tube for inert gas purging (see Experimental Section).

The right branch of the MCP depended on the pretreatment of the Au electrode. The $C_{\text{ACV}}$ data in Figure 3D shows the right branch of M3 is significantly more leftward than that of M1 or M2. An upper vertex potential of only 1.1 V versus RHE introduced changes to the surface that affect the MCP of M1 and M2. In the literature, gold specimens are often pretreated with CV using vertex potentials way above 1.1 V versus RHE or flame annealing, aiming to clean the surface.^[^ ^36^, ^78^, ^79^ ^]^ Such pretreatments affect the surface oxidation and thus the right branch of the measured MCPs. The right branch of the measurement M3 shown in Figure 3D represents a peak that caused a camel‐type MCP that is also often reported in the literature.^[^ ^23^, ^36^ ^]^ The related peak may indicate a change of oxdiation or a switch of oxide states from a conductive to a less conductive species that decreases the electrode capacitance.^[^ ^63^ ^]^ However, attributing this peak to intrinsic potential dependencies of DL capacitances is not reasonable when the effects of the oxide formation are ignored.

Concluding, between 0.1 and 0.8 V, the MCP of a freshly polished gold electrode in argon‐purged electrolyte is shown as mostly flat. The electrode and electrolyte pretreatment significantly affect the MCP and can be responsible for many of the valley‐ or camel‐type features observed in the literature.^[^ ^18^, ^20^, ^23^ ^]^ A certain amount of influence of the intrinsic DL properties on the capacitance especially for the right branch of the MCP cannot be excluded, which is also responsible for the unsymmetric CV data typically observed with gold electrodes.^[^ ^59^ ^]^ However, a clear parabola‐like MCP in the region of the PZC could not be observed.

### 3.5 The Role of the Electrode Material

As reviewed in the “Introduction”, historically, the measurement and theory development of the DL started with dropping mercury electrode. This electrode enables capillary and electric measurements to extract capacitances. The thermodynamic data of Pourbaix^[^ ^70^ ^]^ shows that the equilibrium potential for mercury oxidation is 0.84 V with respect to an amount of 1 μM dissolved mercury ions at neutral pH. Like the gold electrode, the surface oxidation of the mercury electrode is expected to start at lower potentials than that of the thermodynamic standard potential. More importantly, the case of gold showed the electrode history and previous oxidation significantly affects the surface and reduction‐resistant oxide species. Unlike the case of gold, oxygen from the air or the electrolyte can directly react with the less noble mercury surface, displaying an additional factor that influences capacitance measurements. Hence, the electric capacitance measurements on the mercury electrode are affected by more systematic errors than those of the gold electrode.

The electric and electrocapillary measurements of mercury roughly correlate.^[^ ^17^, ^19^, ^20^ ^]^ Bubble evolution and oxide layer formation also affect the surface tension. The polarity of the oxide increases the ability of mercury to wet a glass surface. Different electron affinities of adsorbed hydrogen and the mercury surface also lead to dipoles that change the wetting behavior. Hence, the electrocapillary and electric capacitance measurements are expected to react similarly to the influence of HER, ORR, and oxidation. Moreover, the parasitic effects on the capacitance may also directly influence the electrocapillary by the Lippman equation.^[^ ^16^ ^]^ The parabola‐, bell‐, or camel‐type MCPs at mercury were often related to the DL properties,^[^ ^19^, ^20^, ^80^ ^]^ neglecting any other physicochemical effects that contributed to these measurements.

The classical DL models used to calculate the DCAP do not include the formation of the oxide layers and their potential dependencies. However, as discussed above for the case of mercury and gold, the oxidation processes significantly affect the MCPs. **Table** **1** shows reported PZCs of transition metals as extracted by the minimum in MCPs.^[^ ^12^ ^]^ Moreover, the standard potentials (STPs) of these metals are shown in Table 1. The following metals have a similar (±0.1 V) or higher PZC than the STD: Al, Bi, Cr, Fe, Ga, Nb, Ni, Pb, Ta, Ti, and Zn. The values for the PZC for these metals are obviously affected by oxide layers and are thus not relatable to the intrinsic properties of the DL.

**Table 1:** PZCs versus NHE from ref. [12] and STP versus the SHE from ref. [66] for various metals (the potentials of both reference electrodes roughly correlate). Some metals show a higher PZC than the STPs, indicating a falsification of the PZC determination by oxide layers. *: no data available.

Metal	PZC (vs. NHE)	STP (vs. SHE)
Ag	−0.44	0.8
Al	−0.52	−2.33
Au	0.18	1.52
Bi	−0.39	−0.8
Cd	−0.72	−0.4
Co	−0.45	−0.28
Cr	−0.45	−0.74
Cu	0.09	0.34
Fe	−0.35	−0.44
Ga	−0.69	−0.55
Hg	−0.19	0.79
In	−0.65	−0.34
Ir	−0.04	*
Nb	−0.79	−1.1
Ni	−0.3	−0.26
Pb	−0.62	−0.58
Pd	0	0.92
Pt	0.02	1.19
Rh	−0.02	*
Sb	−0.14	0.2
Sn	−0.43	−0.13
Ta	−0.85	−0.75
Ti	−1.05	−1.31
Tl	−0.75	−0.34
Zn	−0.63	−1.2

Next to gold, the platinum group metals (Pt, Pd, Ru, Ir, Re, Os) have the highest nobleness across the metals in the periodic table. However, in aqueous electrolytes, their capacitances are affected by pseudocapacitances associated with the hydrogen and oxygen adsorption.^[^ ^60^ ^]^ These pseudocapacitances were widely ignored for the PZC estimation,^[^ ^81^, ^82^, ^83^ ^]^ but obviously the minimum of the MCP here just represents the region with least pseudocapacitive hydrogen or oxygen adsorption. In the electrochemical series, mercury and silver display the following next most noble metals. Both metals have similar nobleness and are affected by the above‐described oxidation by atmospheric oxygen and a very narrow window between the electrochemical oxidation and hydrogen evolution reaction. For less noble metals, MCPs mainly consist of competing electrode oxidation and HER. As a result, these MCPs cannot be meaningfully correlated with the DCAPs from models that represent the intrinsic DL properties. Hence, the measured PZCs are consequently not reliable. With reference to the above discussed difficulties of the MCPs of metals, gold displays the only polished metal for which MCPs can be measured in aqueous electrolytes by simultaneously fulfilling 1) an initially nonoxidized surface, 2) no distinct hydrogen or oxygen adsorption (like the platinum group metals), and 3) a broad potential range in which neither the electrode oxidation nor the HER significantly contribute.

The DL of gold was also examined in aprotic solvents,^[^ ^21^ ^]^ where the hydrogen evolution reaction and the electrode oxidation were avoided. However, the formation of the solid electrolyte interphase significantly affects the capacitance and causes a charge transfer reaction,^[^ ^84^ ^]^ which consequently cause nonflat MCPs. Besides the electrical measurements of the PZC, optical measurements to determine the PZC were discussed.^[^ ^85^ ^]^ However, like the electrocapillary of the mercury electrode, it remains questionable whether changes of the surface state such as oxide layers or adsorbed species also impact the optical properties of the surface.

### 3.6 The Role of the Measurement Technique

Historically, MCPs were obtained with a potentiostat in combination with a function generator that added a sinusoidal voltage perturbation. A lock‐in amplifier analyzed the current response of the perturbation regarding the amplitude and phase angle, from which the impedance (and thus capacitance) could be determined.^[^ ^24^ ^]^ However, in this approach, the potentiostat counteracts against the enforced perturbation, displaying a potential error cause that may have falsified reported measurements. As described in the Experimental Section, the data discussed in this study were obtained with a modern‐day potentiostat, which applies the sinusoidal perturbation to the linear potential scan in the time domain. Thus, counteracting signals were avoided. In some recent works,^[^ ^86^, ^87^ ^]^ MCPs were determined by dividing the CV current by the scan rate. In this case, charge transfer reactions and capacitive contribution to the CV cannot be distinguished, for which this kind of measurement is experimentally less precise than the first electric capacitance measurements on the mercury electrode that were collected almost a century ago.^[^ ^17^ ^]^

When probing the DL response, the amount of displaced charge and the associated capacitance increases toward lower frequencies.^[^ ^63^, ^65^ ^]^ This frequency effect led to different values of $C_{\text{EIS}@\text{RF}}$ and $C_{\text{EIS}@1\text{Hz}}$ in Figure 3. The MCPs with $C_{\text{EIS}@1\text{Hz}}$ and $C_{\text{ACV }}$ mostly agreed, as both were measured at the same frequency. CV and EIS data of DL responses are for small amplitudes transferable via a Fourier transformation,^[^ ^88^ ^]^ showing that the form of perturbation has a minor impact on capacitances measured. In contrast to the sinosoidal perturbations, with the step function, the transition between steady states is measured. Hence, for this technique, the dynamics of the ion rearrangement in the DL and the related relaxation are less important, which better portray the situation assumed for the DCAP calculations.

### 3.7 A Critical View on DCAP and PZC Concepts

The classical theories for the DL describe electrostatic scenarios, which display stationary states without a current. The DCAP is typically^[^ ^37^ ^]^ derived by differentiating the charge distribution *ρ* in the electrode potential *E*

(2) $$C_{\text{diff}}=\frac{\partial \rho }{\partial E}$$

Hence, the DCAP describes the transition between static states but does not give any information about the dynamics of the transition. However, the DL response is an electrodynamic process with a n‐body interaction of moving ions by diffusion and electromigration that results in a time‐dependent electric field penetration during the charge rearrangement in the DL.^[^ ^59^, ^64^ ^]^ Hence, interpreting the DL as a capacitance in terms of Equation (2) is an oversimplification. When extracting the capacitance from measurements that used potential variations, the dynamics of the ion rearrangement is measured, which cause resistive contributions that are often taken into accoutn by the constant phase element (CPE) parameterization.^[^ ^57^, ^59^ ^]^ Hence, the commonly used assumption ^[^ ^7^, ^19^, ^22^, ^23^ ^]^ that MCPs display measured DCAPs and even the same wording for both is not correct. The series combination of capacitances described by Equation (1) from Sterns model and the following works does not account for the dynamics of the ion transport in the DL. By presuming Equation (1) and neglecting the DL dynamics, Parsons–Zobel plots^[^ ^89^ ^]^ are commonly used to distinguish between inner and outer Helmholtz layer contributions,^[^ ^29^, ^90^, ^91^ ^]^ which are however not meaningful when the DL dynamics are actually probed in the experiments.

To directly measure the transition between static states that is described by the DCAP with Equation (2), the amperometry‐step‐function procedure introduced above to determine $C_{\text{SQW}}$ may be usable. This technique starts and ends with a steady state (static states with negligible current are due to the parasitic reactions never reachable), and the dynamics in between this relaxation do not matter when the total charge of the transition (like Equation (2)) is counted. However, charge transfer reactions directly affect the measured currents and the capacitances $C_{\text{SQW}}$ therefrom calculated.

### 3.8 Assumptions of the Electrostatic DL Models

The electrostatic models for the DL assume a static statistical distribution^[^ ^37^ ^]^ (lattice gas model or Boltzmann) of the ions in the electrolyte, while the electrochemical interface is assumed as a macroscopic electrostatic equilibrium without moving ions at a constant electrode potential of a blocking electrode (no parasitic currents). However, the attraction of contrary and the distraction of evenly charged ions in combination with the Brownian motion lead to continuous ion rearrangements that interact with electrodynamic forces. Hence, the electrolyte and the DL are in a dynamic state rather than in a static electrostatic order. Consequently, the mean‐field assumptions used for the Poisson–Boltzmann framework^[^ ^38^, ^92^ ^]^ are not justifiable, while the microscopic dynamics are ignored in the electrostatic models that assume a macroscopic equilibrium. Similar criticism of the Deby–Hückel theory for electrolytic solution was recently published,^[^ ^93^ ^]^ which relies on a similar Poisson–Boltzmann framework as the Gouy*–*Chapman theory. The mathematical independence of states presumed for the Boltzmann statistics is violated by convolution with the Poisson equation.^[^ ^93^ ^]^ Moreover, the assumption of a dielectric continuum on an atomistic level is not justified,^[^ ^50^ ^]^ so the dipoles of the solvent are not adequately represented in the classical theories.^[^ ^93^ ^]^ The definition of a conductive dielectric in an electrostatic framework is also erroneous with reference to the electric field‐free conductors in classical electrostatics that was discussed in the “Introduction.”^[^ ^93^ ^]^

To summarize, the DL displays a dynamic state of matter and electrostatic models with mean‐field assumptions do not portray the spatially and temporally varying atomistic and molecular interactions. By adding multiple extensions to the electrostatic theories^[^ ^25^, ^26^, ^27^, ^38^, ^39^, ^40^, ^41^, ^42^ ^]^ an overfitting of the theory to MCPs resulted, which probes the dynamic charge rearrangement in the DL rather than the transition between static states that is calculated with the DCAP. Consequently, the 100‐year‐old electrostatic models that are the basis of our today's textbook knowledge about the DL should be considered with care and skepticism. This study aims to raise awareness for such long‐overlooked inconsistencies of the electrostatic DL theories.

## 4 Conclusions

In this study, MCPs on a polycrystalline gold electrode are critically examined regarding the effect of oxide layer formation/reduction, hydrogen evolution reaction, and oxygen reduction reaction. By varying the examined potential windows and the amount of dissolved electrolytic oxygen, the contributions of the oxide formation/reduction and oxygen reduction reaction are controlled. This variation is shown to significantly affect the MCPs as charge transfer processes directly affect capacitances. The gold electrode shows a capacitance enlargement by the initial oxide layer formation, which irreversibly changes the MCPs toward the valley or camel‐type shape that was reported for previously oxidized gold specimens. By probing the capacitance at relaxation frequencies, the contributions of parasitic reactions to the response are weakened, resulting in almost flat capacitance profiles for non‐ or weakly‐oxidized gold. The DCAP from theory was discussed to describe a transition between static states, which does not describe the dynamic ion rearrangement of the ions in the DL that is actually measured. MCPs of other metals than gold are discussed as more vulnerable to nonintrinsic DL contributions, such as pseudocapacitances or oxide formation/reduction. The minima of MCPs are often associated with PZCs, which however cannot be determined with a rather flat MCP or dominant contributions of parasitic reactions. These findings hopefully encourage the community to critically assess electrostatic textbook models of the DL as well as PZCs and MCPs as deceptive evaluation metrics.

## Conflict of Interest

The authors declare no conflict of interest.

## Author Contributions

**Maximilian Schalenbach**: Conceptualization, Methodology, Experimental works, Data Curation, Writing—Original Draft, Visualization. **Hermann Tempel**: Writing—review & editing (supporting). **Rüdiger‐A. Eichel**: Writing—review & editing (supporting).

## Supporting information

Supplementary Material

## Data Availability

The data that support the findings of this study are available from the corresponding author upon reasonable request.
