# Supplementary material for: Capacitance Measurements for Evaluating Electrochemical Double‐Layer Models and Potentials of Zero Charge: A Reassessment
Source: Chemphyschem. 2025 May 25;26(14):e202401088. doi: 10.1002/cphc.202401088 (PMC12276039; doi:10.1002/cphc.202401088)
Supplement: Supplementary file 1 — Supplementary Material [file CPHC-26-e202401088-s001.pdf]

Supporting Information: "Capacitance measurements for evaluating electrochemical double layer models and potentials of zero charge - A reassessment"

Maximilian Schalenbach* [a], Hermann Tempel, Rüdiger-A. Eichel[a,b]

[a] Institute of Energy Technologies (IET-1): Fundamental Electrochemistry, Forschungszentrum Jülich, Wilhelm-Johnen-Straße, 52425 Jülich, Germany
[b] Institute of Physical Chemistry, RWTH Aachen University, 52062 Aachen, Germany
* *Corresponding authors: m.schalenbach@fz-juelich.de*

1 Equations to calculate the differential capacitance

The differential capacitance C_{diff} of the electrostatic models is calculated (as in detailled discussed in reference [1]) by the derivative of the modeled charge density ρ after the electrode potential E:

$$C_{\mathrm{diff}} = \frac{d\rho}{dE}. \tag{S1}$$

In the classical Gouy-Chapman theory, C_{diff} is calculated to [1]

$$C_{\mathrm{diff}} = \frac{\varepsilon_0 \varepsilon}{L_{\mathrm{D}}} \cosh \frac{z e_0 (E - E_{\mathrm{PZC}})}{2 k_{\mathrm{B}} T}, \tag{S2}$$

where ε_0 denotes the vacuum permittivity, ε the permittivity of the electrolyte (assumed as 80), k_{B} the Boltzmann constant, T the temperature, E_{PZC} the potential of zero charge, z the valency, and L_{D} the Debye length. The Debye length is calculated by

$$L_D = \sqrt{\frac{\varepsilon_0 \varepsilon k_{\mathrm{B}} T}{2 (ze)^2 c_0}}, \tag{S3}$$

where c_0 denotes the concentration of ions in the solution. The capacitance of Sterns model is then calculated to [1]

$$C_{\mathrm{Stern}} = \left(\frac{1}{C_{\mathrm{diff}}} + \frac{1}{C_{\mathrm{IH}}} \right)^{-1}, \tag{S4}$$

where C_{IH} denotes the inner Helmholtz capacitance. This inner Helmholtz capacitance is calculated by

$$C_{\mathrm{IH}} = \left[\frac{1}{2 \varepsilon_0 \varepsilon} \left(\sigma_i + \frac{\varepsilon - 1}{\lambda} \sigma_s \right) \right]^{-1}, \tag{S5}$$

where σ_i denotes the diameter of the ions (assumed as 10^{-10} m for anions and cations, respectively), σ_s the diameter of the solvent molecules (assumed as 10^{-10} m), and λ the dielectric properties at the interface [1]. The value of λ is calculated by solving the equation $\lambda^2(1 + \lambda^4) = \varepsilon$ numerically [1], yielding a value of 1.468.

In the lattice gas model, the differential capacitance is calculated to [2]

$$C_{\mathrm{diff}} = \frac{\varepsilon_0 \varepsilon}{L_{\mathrm{D}}} \frac{\gamma |\sinh \Psi_0|}{[1 + \gamma(\cosh \Psi_0 - 1)] \sqrt{2 \ln[1 + \gamma(\cosh \Psi_0 - 1)]}}, \tag{S6}$$

with the dimensionless surface potential Ψ_0 that is calculated by:

$$\Psi_0 \;=\; ze_0 \frac{E - E_0}{2k_\mathrm{B}T}\,. \tag{S7}$$

The plots show in the introduction of the article were calculated with $c_0 = 1\ \mathrm{mM}$ and $E_\mathrm{PZC} = 0.3\ \mathrm{V}$.

2 Impedance spectra

Figure S1, S2, and S3 show the recorded impedance spectra for the measurements M1, M2, and M3, respectively. Moreover, the capacitance dispersion that is calculated based on the impedance data is graphed [3,4]. The different colors represent the potential steps. To avoid overloading, the data of every second step is graphed, so that a difference of 0.1 V between succeeding spectra results.

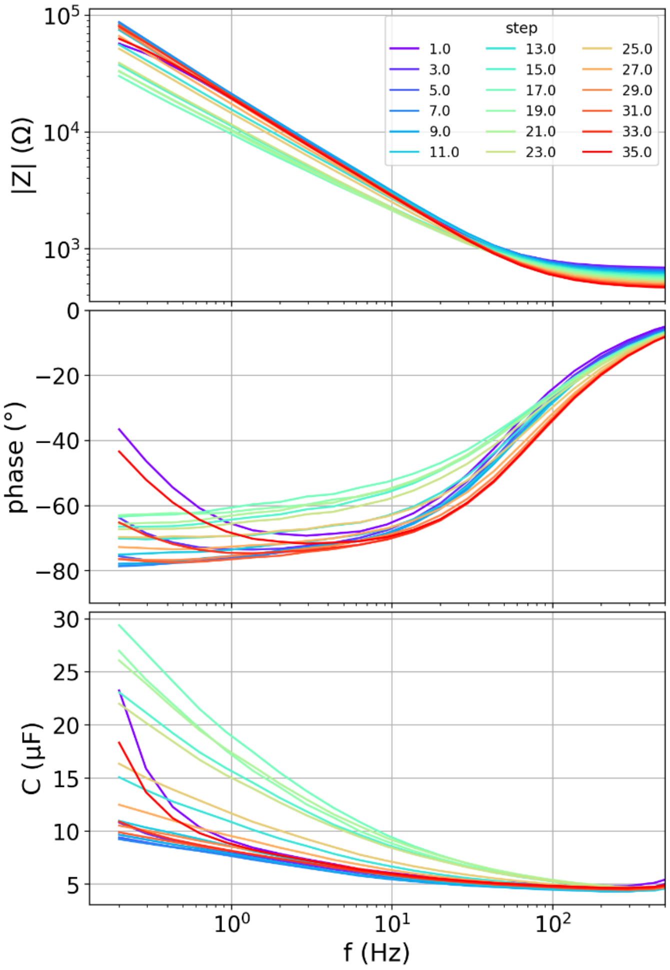

Figure S1: Impedance magnitude, phase angle, and capacitance dispersion recorded during the measurement M1. The impedance spectra of every second potential step is shown.

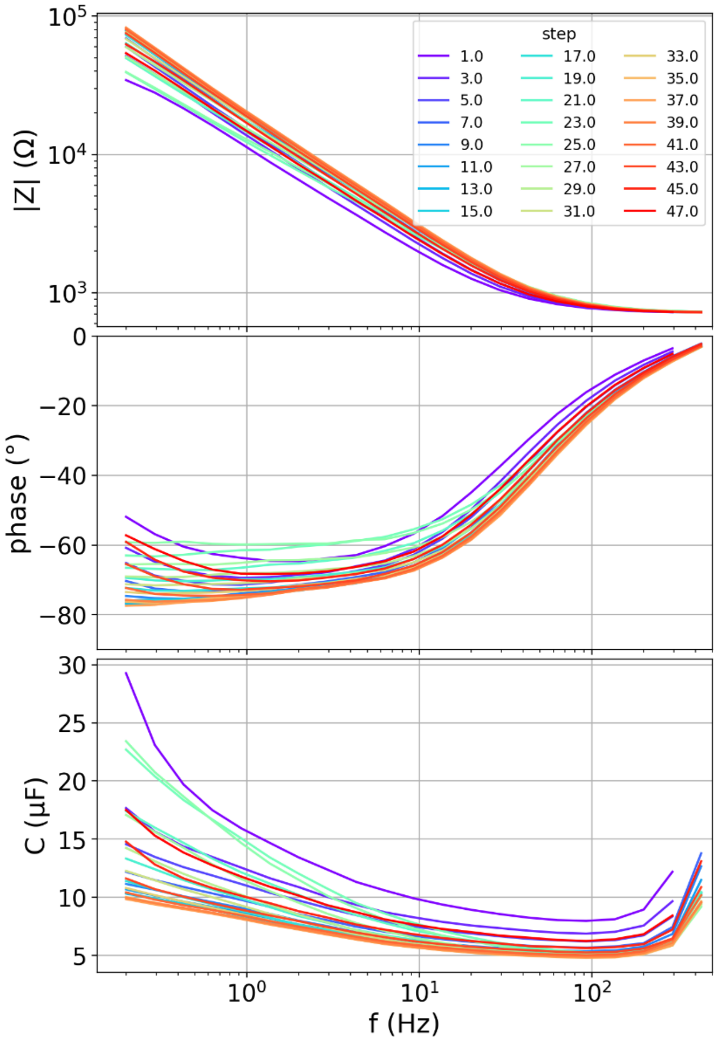

Figure S2: Impedance magnitude, phase angle, and capacitance dispersion recorded during the measurement M1. The impedance spectra of every second potential step is shown.

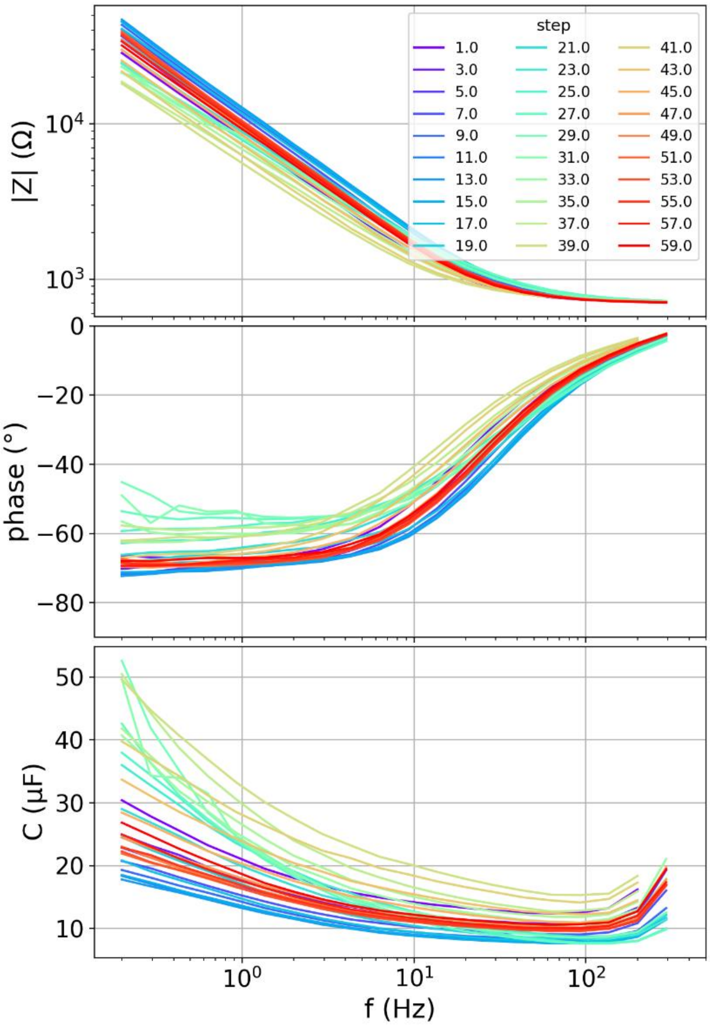

Figure S3: Impedance magnitude, phase angle, and capacitance dispersion recorded during the measurement M1. The impedance spectra of every second potential step is shown.

3 Repetition measurements

Figure S4 shows repetition measurements with the same protocol on the gold electrode.

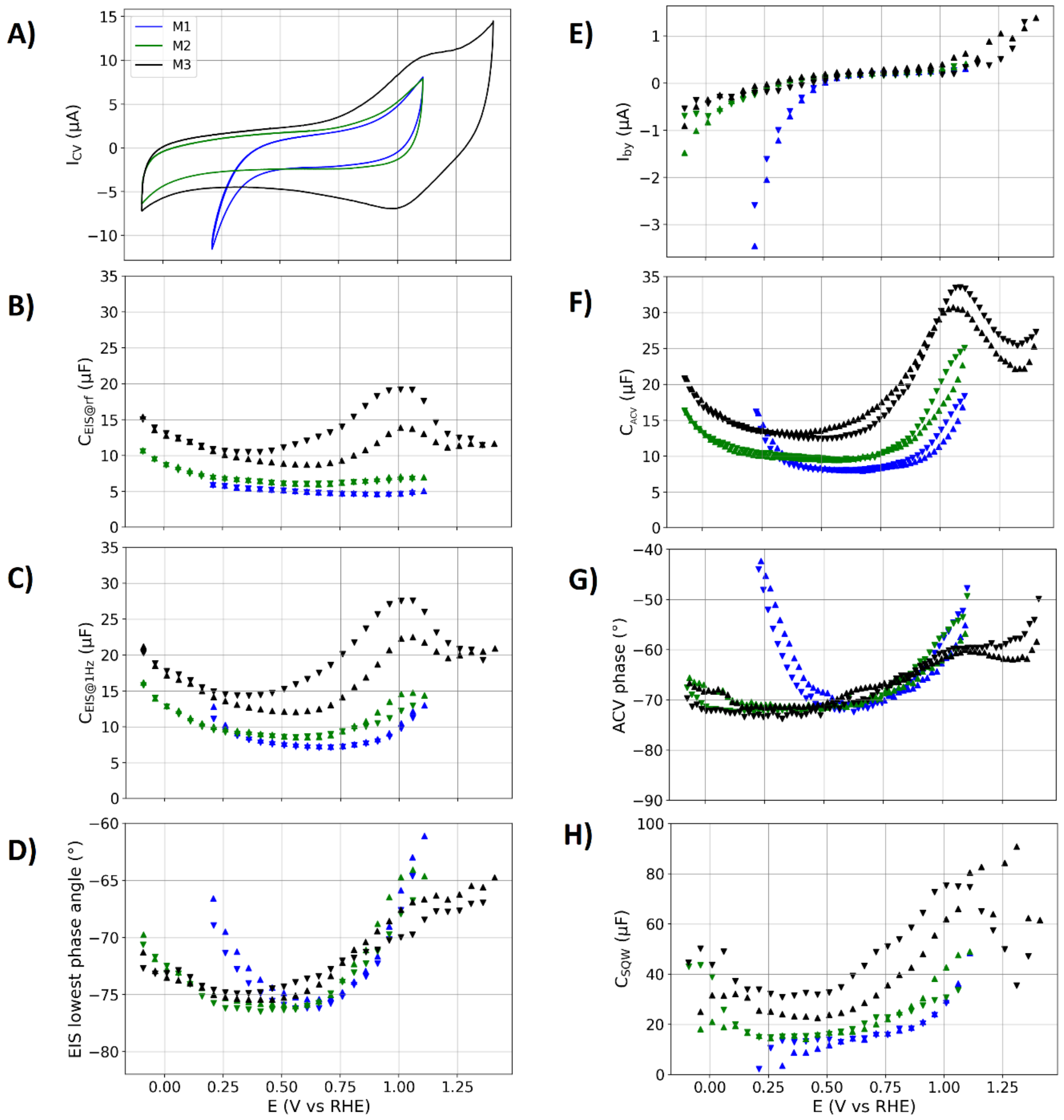

Figure S4: Repetition measurement of the data shown in the article.

4 Python codes

4.1 Code to extract the capacitance and phase angle from ACV data

```
import pandas as pd
import numpy as np

import sys
# insert at 1, 0 is the script path (or '' in REPL)
sys.path.insert(1, 'C:\PythonToolBox')

import CV_Eval

def determine_times_zero_crossings(df_test, array_name,AC_frequency):

    #default values
    minimum_amount_of_data = 1
```

```
    factor_zero_data = 0.2 # to detmeine a zero-crossing, data < 20% of the amp is kept, the rest is set
to 0

    mean_data_record_time = df_test.t.diff().mean()
    number_of_data_points_per_period = 1/(mean_data_record_time*AC_frequency)
    time_period = 1/AC_frequency

    #print(mean_data_record_time)
    #print(number_of_data_points_per_period)

    # between 2 zero-crossing at least "margin_t_window_period" of the time of one period,
otherwise data rejected)
    margin_t_window_period = 0.5/2 # 50% of half a period
    t_window_between_zeros = margin_t_window_period*time_period

    #print(t_window_between_zeros)
    # correct for offset
    df_test["array"] = df_test[array_name] - df_test[array_name].mean()
    df_test["array_abs"] = abs(df_test["array"])
    df_zero = df_test[df_test["array_abs"]<df_test["array_abs"].max()*factor_zero_data]

    # visualize results
    #plt.plot(df_test.t,df_test["array"])
    #plt.plot(df_test.t,df_test["array_abs"])
    #plt.plot(df_zero.t,df_zero["array_abs"])

    # focus on zero crossings
    df_zero["diff_time"] = df_zero["t"].diff()
    df_zero["diff_time_change"] =
df_zero["diff_time"].where(df_zero["diff_time"]<=t_window_between_zeros,1).where(df_zero["diff
_time"]>=t_window_between_zeros,0)
    df_zero["number_crossing"] = df_zero["diff_time_change"].cumsum()

    # Evaluate zero crossings
    dict_slopes = {}
    dict_amount_data = {}
    dict_t_mean = {}

    # detemrine amount of data and slpoes
    # if enough data and if postive slope of the crossing, than detemrine times
    for i in range(0,int(df_zero["number_crossing"].max())+1):
        df_zero_test = df_zero[df_zero["number_crossing"]==i]
        number_data_points = len(df_zero_test)
        #print(number_data_points)
        dict_amount_data[i] = number_data_points
        if number_data_points > minimum_amount_of_data:
            mean_slope = df_zero_test["array"].diff().dropna().mean()
            if mean_slope>0:
                dict_slopes[i] = 1
                dict_t_mean[i] = df_zero_test["t"].mean()
            else:
```

```
            dict_slopes[i] = 0
    return np.asarray(list(dict_t_mean.values()))

def determine_phase(E_zeros,I_zeros,AC_frequency):
    # detemriens the phase angle based on
    t_diff = I_zeros[I_zeros < E_zeros.max()].max()-E_zeros.max()
    phase_pi = (t_diff/(1/AC_frequency))*(2*np.pi)
    phase_deg = phase_pi*360/(2*np.pi)
    return t_diff, phase_deg

def determine_amplitude(df_test,array_name):
    # rudementary, needs further improvement

    return (df_test[array_name].max()-df_test[array_name].min())/2

df_smooth = pd.DataFrame()
E_zeros = []
I_zeros = []
def ACV_evaluation(df_test,window1,window2,AC_frequency,amount_periods):
    #global df_slice
    #global df_smooth
    #global E_zeros
    #global I_zeros
    #show smoothed data
    #window1 = 1000
    #window2 = 5

    # Extract DC by extensive smoothing
    df_smooth = CV_Eval.smooth(df_test,"boxcar",window1)
    df_smooth["E_DC"] = df_smooth["E_smooth"]
    df_smooth["I_DC"] = df_smooth["I_smooth"]

    # reduce noise
    df_smooth = CV_Eval.smooth(df_smooth,"boxcar",window2).copy()

    # Extract smoothed and unsmoothed AC data
    df_smooth["E_AC"] = df_smooth["E"]-df_smooth["E_DC"]
    df_smooth["I_AC"] = df_smooth["I"]-df_smooth["I_DC"]

    df_smooth["E_AC_smooth"] = df_smooth["E_smooth"]-df_smooth["E_DC"]
    df_smooth["I_AC_smooth"] = df_smooth["I_smooth"]-df_smooth["I_DC"]

    df_smooth["t"] = df_smooth["t"] - df_smooth["t"].min()


    df_results = pd.DataFrame(columns =
["E_DC","I_DC","E_zeros","I_zeros","E_Amp","I_Amp","t_diff","phase"])

    # definitions of times
    slice_time = (1/AC_frequency)*amount_periods
    total_time = df_smooth["t"].max() - df_smooth["t"].min()
```

```
    for i in range(0,int(total_time/slice_time)):
        df_slice = df_smooth[(df_smooth["t"] >= i*slice_time) &  (df_smooth["t"] <
slice_time*(i+1))].copy()

        if len(df_slice) < 5:
            print("too short")
        # determine phase
        E_zeros = determine_times_zero_crossings(df_slice,"E_AC_smooth",AC_frequency)
        I_zeros = determine_times_zero_crossings(df_slice,"I_AC_smooth",AC_frequency)

        t_diff, phase = determine_phase(E_zeros,I_zeros,AC_frequency)

        # detemrine amplitude
        E_Amp = determine_amplitude(df_slice,"E_AC_smooth")
        I_Amp = determine_amplitude(df_slice,"I_AC_smooth")

        #
        E_DC = df_slice["E_DC"].mean()
        I_DC = df_slice["I_DC"].mean()

        df_results.loc[i] = [E_DC,I_DC,E_zeros, I_zeros, E_Amp, I_Amp, t_diff, phase]

    df_results["Z"] = df_results["E_Amp"]/df_results["I_Amp"]
    df_results["Z''"] = df_results["Z"]*np.sin(df_results["phase"]*2*np.pi/360)
    df_results["Z'"] = df_results["Z"]*np.cos(df_results["phase"]*2*np.pi/360)
    df_results["C"] = -1/(2*np.pi*AC_frequency*df_results["Z''"] )

    return df_smooth, df_results

scan_rate = 0.01 #V/s
```

4.2 Code to extract the capacitacnes at the relaxation frequencies

```
def evaluate_impedance(df):
    # Determine the electrolyte resistance
    df = df.sort_values(by=['f'],ascending = False)
    df = df.reset_index(drop = True)

    # filter frequency range for evaluation
    df = df[df["f"]< 2e5]
    df = df[df["f"]> 0.1]

    # filter phase angle
    df = df[df["phase"]<0]

    # ist ja totaler quatsch!!!
    df_test = df.copy()
    df_test = df_test[df_test["f"]>1e3].copy()
    index_R_s = np.argmin(abs(df_test["phase"]))
    R_s = df_test.at[df_test.index[index_R_s],"Z_real"]

    # determine relaxation frequency
    df_r = df.copy()
```

```
iindex_phase_min = np.argmin(df_r["phase"])
index_phase_min = df_r.index[iindex_phase_min]
df_r = df_r[df_r.index<=index_phase_min]
index_relax = np.argmin(abs(df_r["phase"] +45))
low = index_relax-1
high = index_relax+1
f_relax = np.interp(45, -df_r["phase"].iloc[low:high], df_r["f"].iloc[low:high])
C_relax =  np.interp(45, -df_r["phase"].iloc[low:high], df_r["C"].iloc[low:high])

phase_min = df_r["phase"].min()
index_1Hz = np.argmin(abs(df["f"] -1))
C_1Hz = df.at[df.index[index_1Hz],"C"]

index_10Hz = np.argmin(abs(df["f"] -10))
C_10Hz = df.at[df.index[index_10Hz],"C"]

index_01Hz = np.argmin(abs(df["f"] -0.1))
Z_real_01Hz = df.at[df.index[index_01Hz],"Z_real"]
phase_01Hz = df.at[df.index[index_01Hz],"phase"]
return [R_s,phase_min, f_relax, C_relax*1e6,C_1Hz*1e6,phase_01Hz,Z_real_01Hz]
```

References

1. W. Schmickler, *Electrochemical Theory: Double Layer*, p. 1–10, Elsevier Inc., Reference Module in Chemistry, Molecular Sciences and Chemical Engineering, (2014).
2. S. May, *Curr. Opin. Electrochem.*, **13**, 125–131 (2019).
3. M. Schalenbach, L. Raijmakers, V. Selmert, A. Kretzschmar, Y. E. Durmus, H. Tempel, and R.-A. Eichel, *Phys. Chem. Chem. Phys.*, **20**, 1 (2024).
4. M. Schalenbach, Y. E. Durmus, S. Robinson, H. Tempel, H. Kungl, and R. Eichel, *Phys. Chem. C*, **125**, 5870–5879 (2021).
